# Supplementary material for: Association between Daily Activities and Behavioral and Psychological Symptoms of Dementia in Community-Dwelling Older Adults with Memory Complaints by Their Families
Source: Int J Environ Res Public Health. 2020 Sep 18;17(18):6831. doi: 10.3390/ijerph17186831 (PMC7558144; doi:10.3390/ijerph17186831)
Supplement: Supplementary file 1 [file ijerph-17-06831-s001.pdf]

Table S1.Shopping in PADA-D

Score	Process	actions that make up the process	check		Remarks
	1.Enter the store	(1) Enter the store	YES	NO	
		(2)Take a shopping basket / cart	YES	NO	
		(3)Confirm the section	YES	NO	
	2. Go to the section	(1)Go toward the section	YES	NO	
		(2)Walk without hitting people or product shelves	YES	NO	
		(3)Get to the section	YES	NO	
	3.Find a product	(1)Find the target product	YES	NO	
		(2)Check the price and expiration date	YES	NO	
		(3)Put the required quantity of the products in the basket	YES	NO	
	4.Pay for the product	(1)Stand in line at the cash register	YES	NO	
		(2)Understand and pay the total amount	YES	NO	
		(3)Check the change	YES	NO	
	5.Take home the product	(1)Pick up the product	YES	NO	
		(2)Put the product into a shopping bag	YES	NO	
		(3)Go toward the exit	YES	NO	

Table S2 .Cooking in PADA-D

Score	Process	actions that make up the process	check		Remarks
	1.Plan a meal	(1)Search for and understand the steps for cooking	YES	NO	
		(2)Prepare the ingredients	YES	NO	
		(3)Prepare the cooking utensils	YES	NO	
	2.Prepare the food (wash, cut and cook the ingredients)	(1)Wash the foodstuffs	YES	NO	
		(2)Slice/cut /mash the foodstuffs	YES	NO	
		(3)Fry/ steam/ boil /bake the foodstuffs	YES	NO	
	3.Season the ingredients (choose seasoning, et al)	(1)Choose appropriate seasoning	YES	NO	
		(2)Add a suitable amount of seasoning	YES	NO	
		(3)Check the taste	YES	NO	
	4.Plate the food	(1)Select the appropriate dishes for the food	YES	NO	
		(2)Prepare dishes for the number of people	YES	NO	
		(3)Serve the proper quantity of meal on the dishes	YES	NO	
	5.Set the table	(1)Carry dishes to the table	YES	NO	
		(2)Set dishes on the table	YES	NO	
		(3)Set chopsticks / spoons and fork or knife	YES	NO	

Table S3. Housekeeping in PADA-D

Score	Process	actions that make up the process	check		Remarks
	1. Clean up after a meal	(1)Wash the dishes and cooking utensils	YES	NO	
		(2)Dry the dishes	YES	NO	
		(3)Put the dishes back where they belong	YES	NO	
	2. Managing daily necessities	(1)Understand about the contents of the refrigerator	YES	NO	
		(2)Sort out the mail and the bills	YES	NO	
		(3)Change clothes according to the season	YES	NO	
	3. Management of bedding	(1)Make the bed	YES	NO	
		(2)Change the bedspread regularly	YES	NO	
		(3)Dry the bedding in a dryer	YES	NO	
	4. Clean the house	(1)vacuum and sweep the room	YES	NO	
		(2)Wipe and scrub the room	YES	NO	
		(3)Tidy up the room / put something back	YES	NO	
	5. Garbage dumping	(1)Separate the garbage	YES	NO	
		(2)Put out the garbage on collection day	YES	NO	
		(3)Take the garbage to the dumpster	YES	NO	

Table S4. Use modes of transportation in PADA-D

Score	Process	actions that make up the process	check		Remarks
	1. Take a taxi	(1)Call or catch a taxi	YES	NO	
		(2)Tell the driver the destination	YES	NO	
		(3)Get out at the destination	YES	NO	
	2.Take a bus or train	(1)Go to the station / bus stop	YES	NO	
		(2)Take a train or bus to the destination	YES	NO	
		(3)Get off at the station / bus stop	YES	NO	
	3. Ride a bicycle	(1)Riding a bicycle	YES	NO	
		(2)Obey traffic rules	YES	NO	
		(3)Arrive at the destination and park the bike in a parking lot	YES	NO	
	4. Drive a mobility scooter	(1)Drive a mobility scooter	YES	NO	
		(2)Obey traffic rules	YES	NO	
		(3)arrive at the destination and park the mobility scooter in a parking lot	YES	NO	
	5.Choose an appropriate mode of transportation	(1)Understand the time /distance to the destination	YES	NO	
		(2)Understand the fare for the chosen mode of transportation	YES	NO	
		(3)Use different modes of transportation depending on the situation	YES	NO	

Table S5. Laundry in PADA-D

Score	Process	actions that make up the process	check		Remarks
	1. Put the laundry in the washing machine	(1)Separate the laundry	YES	NO	
		(2)Open the lid of the washing machine	YES	NO	
		(3)Put the laundry into the washing machine	YES	NO	
	2.Start the washing machine	(1)Put some detergent in the washing machine	YES	NO	
		(2)Close the lid of the washing machine	YES	NO	
		(3)Turn on the power and start the washing machine	YES	NO	
	3.Operate the dryer or find another effective means to dry the laundry	(1)Take the laundry out of the washing machine	YES	NO	
		(2)Stretch the wrinkle on the laundry	YES	NO	
		(3)Hang the laundry / put the laundry in the dryer	YES	NO	
	4. Take in and fold the laundry	(1)Check whether the laundry is dry or damp	YES	NO	
		(2)Take in the laundry	YES	NO	
		(3)Fold the laundry according to the shape of clothes	YES	NO	
	5.Put the clothes in the chest/closet	(1)Carry the clothes to the chest/closet	YES	NO	
		(2)Put the clothes in appropriate storage	YES	NO	
		(3)Close drawers and closet door	YES	NO	

Table S6. Managing finances in PADA-D

Score	Process	actions that make up the process	check		Remarks
	1.Handle cash	(1)Take out the required number of coins	YES	NO	
		(2)Take out the required number of bills	YES	NO	
		(3)Get the correct amount of change	YES	NO	
	2.Use cash on a daily life	(1)Mainly handle cash for purchasing of grocery	YES	NO	
		(2)Handle cash for rent and bill payments	YES	NO	
		(3)Handle cash for special events	YES	NO	
	3.Understand household express	(1)Understand the necessary amount for living expenses	YES	NO	
		(2)Understand the amount of savings	YES	NO	
		(3)Understand assets	YES	NO	
	4.Use the bank and the post office	(1)Understand where the seals / passbooks / cards have been stored	YES	NO	
		(2)Understand the procedures for transactions at the bank counter (for withdrawals, deposits, bank transfers/payment)	YES	NO	
		(3)Use the ATM for withdrawals/deposits/ bank transfers/payments	YES	NO	
	5.Use electronic money	(1)Tap card against the card reader	YES	NO	
		(2) Transfer some cash to the card	YES	NO	
		(3)Understand the balance on the card.	YES	NO	

Table S7. Managing medication in PADA-D

Score	Process	actions that make up the process	check		Remarks
	1.Keep the regular time to take medicate	(1)Understand the regular time during the day to take medication	YES	NO	
		(2)Understand the regular time during the day to take medication when outside the home	YES	NO	
		(3)Understand the way of taking the time to remember own medication	YES	NO	
	2.Take out the prescribed medicine	(1)Understand the place to store the medicine	YES	NO	
		(2)Take out the storage case for regular medicine	YES	NO	
		(3)Open the storage case for regular medicine	YES	NO	
	3.Check the correct quantity of medicine	(1)Understand the shape of the medicine	YES	NO	
		(2)Understand the color of the medicine	YES	NO	
		(3)Understand the dosage of medicine	YES	NO	
	4.Take medicine correctly	(1)Take the medicine with water	YES	NO	
		(2)Place a medical patch on the affected part	YES	NO	
		(3)Put ointment on the affected part	YES	NO	
	5.Keep track of leftover medicine	(1)Understand what medicine to take at the next time	YES	NO	
		(2)Understand the shortage /surplus of medicine	YES	NO	
		(3)Self-handling of medicine shortage and surplus	YES	NO	
